# Interleukin-8 and clinical symptoms can be prognostic indicators for advanced cancer patients with cachexia

**DOI:** 10.20407/fmj.2018-022

**Published:** 2020-03-25

**Authors:** Miyo Murai, Takashi Higashiguchi, Akihiko Futamura, Hiroshi Ohara, Norimasa Tsuzuki, Yoshinori Itani, Takaaki Kaneko, Takeshi Chihara, Kan Shimpo, Naomi Nakayama

**Affiliations:** 1 Department of Surgery and Palliative Medicine, Fujita Health University, School of Medicine, Toyoake, Aichi, Japan; 2 Department of Medical Technology, Clinical Examination Division, Fujita Health University Nanakuri Memorial Hospital, Tsu, Mie, Japan; 3 Fujita Memorial Nanakuri Institute, Fujita Health University, Tsu, Mie, Japan; 4 The University of Shimane, School of Nursing and Nutrition, Matsue, Shimane, Japan

**Keywords:** Advanced cancer, Prognostic factors, Cytokines, Cachexia

## Abstract

**Objectives:**

Prognostic prediction is a significant tool for selecting appropriate treatment in advanced cancer patients with cachexia, at a time when it is important to offer high-quality palliative care and improve quality of life until death. In this retrospective study, we investigated the prognostic potential of serum cytokine level and various clinical symptoms by analyzing the pathological conditions and metabolic dynamics of cachexia in advanced cancer patients.

**Methods:**

One hundred and fifty-three advanced cancer patients who underwent palliative care and died at the Department of Surgery and Palliative Medicine, Fujita Health University Nanakuri Memorial Hospital between 1 January 2004 and 30 June 2007 were eligible for the study. We simultaneously assessed their blood factors and clinical symptoms at admission. All patients were divided into two groups according to median survival time to analyze the risk factors for prognosis.

**Results:**

Multivariate analysis revealed the following independent prognostic factors: interleukin (IL)-8 (odds ratio [OR]=4.17, 95% confidence interval [CI]=1.52–11.41, *p*=0.002), general fatigue (OR=1.22, 95%CI=1.03–1.45, *p*=0.019), anorexia (OR=1.19, 95%CI=1.04–1.37, *p*=0.008), dyspnea (OR=1.19, 95%CI=1.02–1.38, *p*=0.024), depression (OR=1.28, 95%CI=1.11–1.47, *p*<0.001), nausea (OR=1.25, 95%CI=1.05–1.48, *p*=0.007), dry mouth (OR=1.19, 95%CI=1.01–1.40, *p*=0.032), and overall assessment score (OR=1.05, 95%CI=1.02–1.09, *p*<0.001). Patients with low IL-8 (<1.347 pg/ml) and low overall assessment score (<26) had significantly better prognosis (both *p*<0.0001).

**Conclusions:**

High IL-8 level and clinical symptoms can be prognostic indicators for advanced cancer patients with cachexia.

## Introduction

In 2011, cancer overtook heart disease as the leading cause of death worldwide. Currently, one in two people will develop cancer, and one in three people will die of cancer. Prognostic prediction is important for improving the quality of life of advanced cancer patients, and can assist with proactive development of preventative measures and radical treatments. Prognostic prediction is a significant tool for medical professionals when selecting appropriate treatments for their patients, and for patients and their families when altering their lifestyle as they face death. Several studies have been conducted on prognostic factors, from an early report in 1972 by Parkers et al.^1^ to a more recent report in 2018.^2^ Maltoni and colleagues analyzed 38 previous studies to determine prognostic factors for advanced cancer patients, and reported strong correlations between prognosis and clinical prediction of survival rate, performance status, anorexia, weight loss, dysphagia, dry skin, dyspnea, delirium, leukocytosis, lymphocytopenia, C-reactive protein (CRP), and palliative prognostic score.^3–5^ Other reports describe the potential utility of interleukin (IL)-6, IL-7, IL-8, and interferon (IFN)-γ as prognostic factors.^6–11^ Furthermore, Lippitz^12^ reported strong correlations of IL-6 and IL-10 with prognosis.^12^

However, to our knowledge, no studies have analyzed the pathological conditions of advanced cancer patients and the metabolic dynamics of cachexia. In the present study, we analyzed the potential predictive value of serum cytokine level and specific clinical symptoms in advanced cancer patients, particularly those during cachexia induced by cancer progression.

## Patients and Methods

We performed a retrospective study using a database of patients who were admitted to and underwent palliative care at the Department of Surgery and Palliative Medicine, Fujita Health University Nanakuri Memorial Hospital between 1 January 2004 and 30 June 2007. All participants were provided with detailed information regarding the study by the principal researcher and were guaranteed safe storage of their data. The ethical aspects of the study were carefully monitored by the research group and approved by the Institutional Review Board of Fujita Health University (HM 16-401).

We simultaneously obtained blood samples and assessed the clinical symptoms of advanced cancer patients at admission to the hospital. The following parameters were measured in the blood samples: IL-6, IL-8, IL-10, tumor necrosis factor (TNF)-α, serum albumin (Alb), CRP, total lymphocyte count (TLC), transthyretin (TTR), retinol-binding protein (RBP), and transferrin (Tf). For some parameters, commercially available enzyme-linked immunosorbent assay (ELISA) kits were used: Human IL-6 ELISA Ready-SET-Go (eBioscience, San Diego, CA, USA); IL-8 Human ELISA Kit (Invitrogen, Camarillo, CA, USA); Human IL-10 ELISA Ready-SET-Go (eBioscience); and TNF-α human ultrasensitive ELISA Kit (Invitrogen).

**Figure 1:**
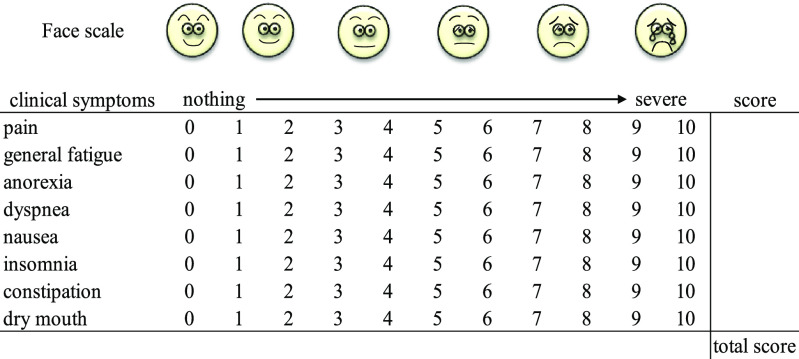
Overall assessment score.

The following nine clinical symptoms were assessed using a face scale^13^ and a numerical rating scale^14^ with 11 possible grade scores (0–10): pain, general fatigue, anorexia, dyspnea, depression, nausea, insomnia, constipation, and dry mouth. The total scores for these nine items were added together to create a comprehensive indicator, designated the overall assessment score (Figure 1). The assessment of clinical symptoms was reorganized by reference to the Edmonton Symptom Assessment System (ESAS-r).^15^ In this study, the clinical symptoms were fundamentally determined by subjective assessment by the patients, but the doctors in charge could provide assistance when patients had difficulty in performing the assessment.

We defined survival time (=prognosis) as the period from blood sampling to death. Potential prognostic factors were assessed using odds ratio (OR) and 95% confidence interval (CI) obtained by logistic regression analyses after dichotomization into short and long median survival time (MST). Factors with a significant influence on poor prognosis in univariate analysis were included in a multivariate logistic regression analysis to determine their adjusted OR. First, univariate logistic regression analyses were performed to identify correlations between prognosis and individual parameters: factors with significant differences in patient characteristics; cytokines (IL-6, IL-8, IL-10, and TNF-α); blood biochemistry indicators (Alb, CRP, TLC, TTR, RBP, and Tf); clinical symptoms (pain, general fatigue, anorexia, dyspnea, depression, nausea, insomnia, constipation, and dry mouth); and overall assessment score. Second, multivariate logistic regression analysis was carried out in a model that included each cytokine with a significant influence in the univariate analysis added to confounding variable blood biochemistry factors, and a model that included each clinical symptom with a significant influence in the univariate analysis added to confounding variable blood biochemistry factors.

### Statistical analysis

Differences were considered significant for values of *p*<0.05. All statistical and data analyses were performed using JMP version 13.0 software (SAS, Cary, NC, USA). Continuous variables were calculated using the Mann–Whitney *U* test; categorical variables were calculated using Fisher’s exact test; survival curves were drawn with Kaplan–Meier curves; and survival rates were compared with the log-rank test.

## Results

The characteristics of the patients are shown in Table 1. The study included 153 advanced cancer patients (90 male and 63 female), with a mean age of 71.5±12.2 years. MST was 51 days. The patients were classified by cancer type as follows: lung cancer, 39 (25.5%); hepatobiliary/pancreatic cancer, 22 (14.4%); gastric cancer, 20 (13.1%); colorectal cancer, 17 (11.1%); renal/urinary tract cancer, 13 (8.5%); breast cancer, 11 (7.2%); head and neck cancer, 11 (7.2%); cranial nerve tumors, 7 (4.6%); cancer of the uterus/adnexa, 4 (2.6%); esophageal cancer, 2 (1.3%); and other, 7 (4.6%).

All patients were divided into two groups according to MST to analyze the prognostic risk factors because all enrolled patients died and poor prognosis could not be identified for terminal stage cancer patients. We defined patients with poor prognosis who died within 51 days (MST) as the short group. We defined patients with relatively good prognosis who survived at least 51 days as the long group.

Comparison of parameters between the short and long groups, and the results of the univariate and multivariate analyses are presented in Table 2. There were significantly more male patients in the short group than long group (*p*=0.016), but age and diagnosis did not differ significantly (*p*=0.673, *p*=0.213, respectively). IL-6, IL-8, IL-10, and TNF-α were analyzed after logarithmic conversion because their data were biased. In the short group as compared with the long group, although the serum levels of IL-6 and IL-8 were significantly higher (1.6 vs. 1.3 pg/ml, p<0.001, 1.6 vs. 1.1 pg/ml, p<0.001, respectively), serum levels of Alb and TTR were significantly lower (2.8 vs. 3.3 g/dl, *p*<0.001, 8.6 vs. 13.6 mg/dl, *p*=0.017, respectively). Various clinical symptoms including general fatigue (*p*<0.001), anorexia (*p*=0.006), dyspnea (*p*=0.004), depression (*p*<0.001), nausea (*p*=0.012), and dry mouth (*p*=0.013) were significantly worse in the short group than in the long group. Overall assessment score in the short group was significantly higher than in the long group (*p*<0.001). As the result of these data, we subsequently performed a multivariate analysis with adjustment for Alb, CRP, and TTR, which showed significant differences in the univariate analyses.

Comparisons of prognosis was based on each significant prognostic factor in multivariate analysis. The following were independent prognostic factors: serum level of IL-8 (OR=4.17, 95% CI=1.52–11.41, *p*=0.002), general fatigue (OR=1.22, 95% CI=1.03–1.45, *p*=0.019), anorexia (OR=1.19, 95% CI=1.04–1.37, *p*=0.008), dyspnea (OR=1.19, 95% CI=1.02–1.38, *p*=0.024), depression (OR=1.28, 95% CI=1.11–1.47, *p<*0.001), nausea (OR=1.25, 95% CI=1.05–1.48, *p*=0.007), dry mouth (OR=1.19, 95% CI=1.01–1.40, *p*=0.032), and overall assessment score (OR=1.05, 95% CI=1.02–1.09, *p*<0.001). Among the other significant prognostic factors, IL-8 and overall assessment score were considered to be the most useful factors to predict patient prognosis.

**Figure 2:**
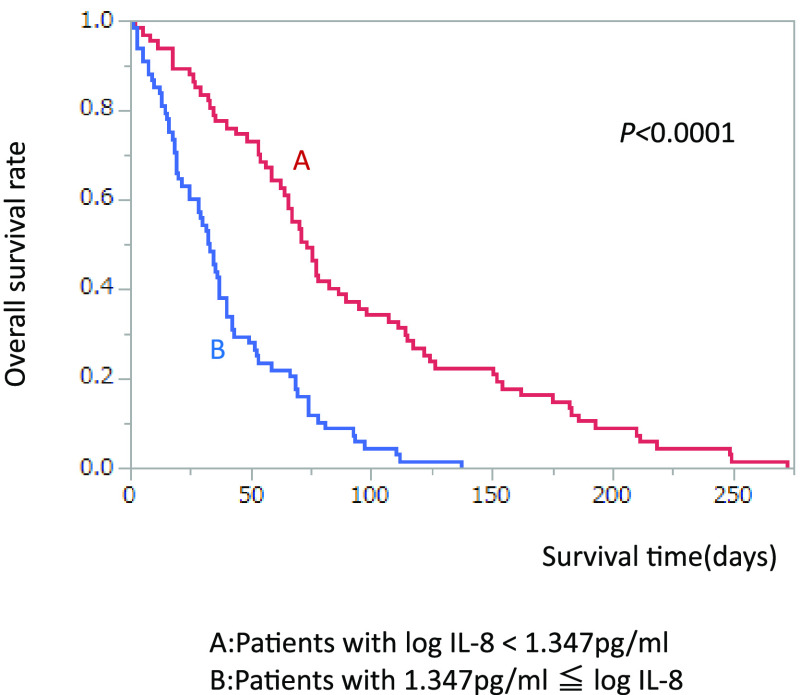
Comparison of survival rate divided by median log IL-8 level.

We divided patients by median level of log IL-8 (1.347 pg/ml). MST in patients with low IL-8 (<1.347 pg/ml) was significantly better than in those with high IL-8 (≥1.347 pg/ml) (73 vs. 32.5 days, *p*<0.0001) (Figure 2).

**Figure 3:**
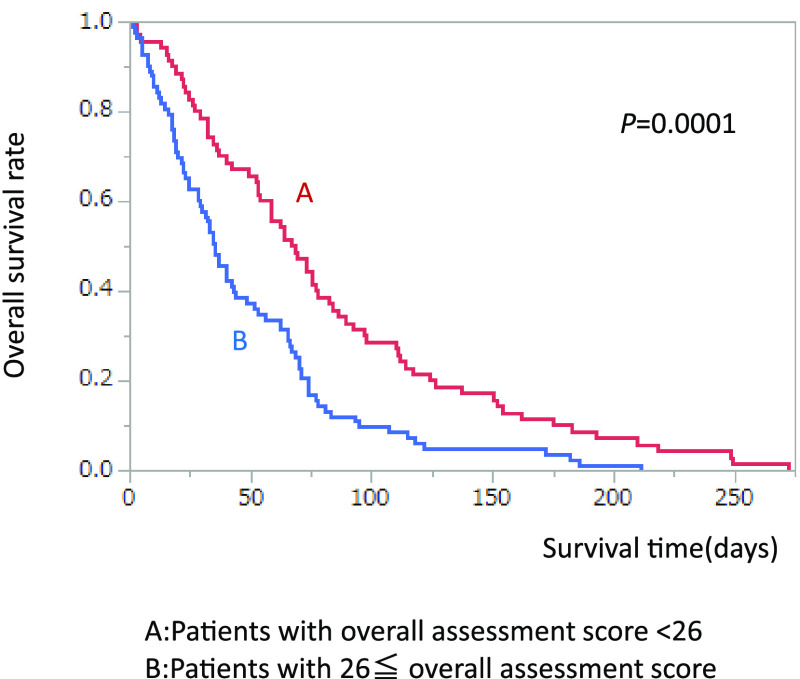
Comparison of survival rate divided by median overall assessment score.

When all patients were divided by median score of overall assessment, MST in the patients with overall assessment score ≥26 was significantly shorter than in patients with overall assessment score <26 (35.0 vs. 67.5 days, *p*<0.0001) (Figure 3).

## Discussion

This study searched for prognostic factors in cancer based on the pathological conditions and metabolic dynamics of cancer cachexia patients. Various metabolic and nutritional disorders develop in cancer patients, and the resulting factors combine to produce complex conditions that present unique challenges for optimal management. Several studies have reported prognostic factors in advanced cancer patients; however, to our knowledge, no reports describe analyses of the pathological conditions and the metabolic dynamics of cachexia. In the present study, we analyzed the potential predictive value of serum cytokine level and specific clinical symptoms in advanced cancer patients; particularly those during cachexia induced by cancer progression.

The European Palliative Care Research Collaborative guidelines for cancer cachexia,^16^ published in 2011, define cancer cachexia as “a complex metabolic disorder characterized by a marked loss of muscle tissue in which improvement through the use of conventional nutritional support is difficult. Pathophysiologically, it is characterized by a negative protein and energy balance due to metabolic abnormalities and reduced oral intake.” The reason for this is considered to be that cachexic cancer patients are in a state of systemic inflammation. This state involves hypercytokinemia and neuroendocrine system dysfunction associated with production of inflammatory cytokines from host tissues in response to proteolysis-inducing factors that are released from cancer cells and cause resistance in tumor cells.

Alb, TLC, TTR, RBP, and Tf have been used as nutritional prognostic factors for many years,^17^ and several studies have shown that CRP has a significant positive correlation with poor prognosis.^18^ TTR has been used as a nutritional index for cachexia, because it can reflect subtle changes in protein metabolism when evaluating the response to a change in nutritional support, and for the diagnosis of irreversible cachexia when TTR does not improve following administration of adequate nutritional therapy. TTR, RBP, and Tf can be used to evaluate metabolic changes in the short term. Serum Alb level is used by many clinicians as a screening index for cachexia. TLC reflects the immune status and decreases with the progression of cancer. CRP indicates the degree of inflammatory reactions in cancer patients and is an established prognostic factor.

The most important finding in the present study is that IL-8 can be a useful prognostic factor for advanced cancer patients. IL-8 is an inflammatory cytokine that induces chemotaxis of neutrophils, and like TNF-α, is believed to be an important mediator of inflammation in the body, because it is enhanced by IL-6, which stimulates macrophages and induces acute inflammation. As in previous studies, our study showed that high level of serum IL-8 was predictive of shorter survival time and poor prognosis in advanced cancer patients.

The second important finding is that clinical symptoms had significant correlations with prognosis. Our study showed that high overall assessment score was predictive of shorter survival time and poor prognosis in advanced cancer patients. Previous studies showed that clinical symptoms of anorexia, dyspnea, and delirium were poor prognostic factors in advanced cancer patients. However, there are no other reports on prognostic prediction that comprehensively evaluate these nine clinical symptoms that are often seen in advanced cancer patients. In this study, IL-8 and clinical symptoms were independent significant factors for prognosis, but the relation could not be seen between these two factors.

There were several limitations to this study. First, it was a retrospective study in a single institution. Therefore, the outcomes of the study may not apply to other institutions. Second, assessment of clinical symptoms is better as a non-invasive indicator compared with blood sampling, but with a view to keeping the number of assessment items low and the burden on patients to a minimum, we need to consider such assessment methods more thoroughly. Third, because patients’ consciousness level becomes unstable as they approach death, subjective assessment tends to become difficult, and we need to determine more objective methods to assess clinical symptoms.

At the Department of Surgery and Palliative Medicine in Fujita Health University, we have practiced new palliative medicine based on the metabolic science of advanced cancer patients since October 2003, at a time when little was known about this subject.^19^ We anticipate the development of treatment methods that can suppress the systemic inflammatory reactions and correct the metabolic abnormalities observed during cancer progression. We consider that the best approach is to understand the state of cachexia, and then define the precise conditions of individual patients. We would hope that survival can be extended by providing palliative treatment that suppresses inflammatory reactions and controls various painful symptoms.

In conclusion, high level of serum IL-8 and overall assessment score can be regarded as useful prognostic indicators, and are strongly associated with poor survival for advanced cancer patients with cachexia. Going forward, we expect that the use of such prognostic indicators will be applied to the care of advanced cancer patients, as well as to the standardization of high-quality palliative care.

## Figures and Tables

**Table1:** Patient characteristics

Number of patients	153
Sex (male/female)	90/63
Age, years (mean±SD)	71.5±12.2
Prognosis, days (median)	51
Diagnosis	
Lung cancer	39 (25.5%)
Hepatobiliary/pancreatic cancer	22 (14.4%)
Gastric cancer	20 (13.1%)
Colorectal cancer	17 (11.1%)
Renal/urinary tract cancer	13 (8.5%)
Breast cancer	11 (7.2%)
Head and neck cancer	11 (7.2%)
Cranial nerve tumours	7 (4.6%)
Cancer of the uterus/adnexa	4 (2.6%)
Oesophageal cancer	2 (1.3%)
Other	7 (4.6%)

SD: standard deviation.

**Table2:** Comparison of parameters between short and long groups, and results of univariate and multivariate analyses

Prognosis, days (median)	short group	long group	Univariate		a) Multivariate	
No. of patients	76	77	OR (95%CI)	P value	OR (95%CI)	P value
Sex (male/female)	52/24	38/39	2.22 (1.15–4.30)	0.016	2.10 (0.92–4.79)	0.077
Age, years (range)	72 (21–92)	73 (42–96)				
Cytokine						
IL-6 pg/ml (IQR)	1.6 (1.2–1.8)	1.3 (1.0–1.5)	5.23 (2.14–12.81)	<0.001	1.18 (0.40–3.48)	0.768
IL-8 pg/ml (IQR)	1.6 (1.3–1.8)	1.1 (0.9–1.4)	7.90 (3.13–19.97)	<0.001	4.17 (1.52–11.41)	0.002
IL-10 pg/ml (IQR)	0.5 (0.3–0.8)	0.5 (0.1–0.6)	2.49 (0.88–7.04)	0.07		
TNF-α pg/ml (IQR)	0.4 (0.1–0.7)	0.3 (–0.1–0.6)	2.03 (1.01–4.12)	0.044	2.00 (0.86–4.67)	0.101
Blood biochemistry indicators						
Alb g/dl (IQR)	2.8 (2.5–3.4)	3.3 (2.9–3.7)	0.30 (0.16–0.57)	<0.001		
CRP mg/dl (IQR)	5.5 (2.4–9.5)	1.7 (0.6–3.7)	1.22 (1.10–1.34)	<0.001		
TLC/μL (IQR)	1000 (715–1330)	1140 (760–1725)	0.99 (0.99–1.00)	0.304		
TTR mg/dl (IQR)	8.6 (5.4–11.6)	13.6 (9.1–17.2)	0.95 (0.91–1.00)	0.017		
RBP mg/dl (IQR)	1.9 (1.3–2.4)	2.4 (1.6–3.3)	1.00 (0.89–1.11)	0.928		
Tf mg/dl (IQR)	142 (114–177)	159 (138–189)	1.00 (0.99–1.00)	0.139		
Clinical symptoms						
Pain (mean±SD)	2.9±2.3	3.0±2.6	1.00 (0.87–1.13)	0.919		
General fatigue (mean±SD)	4.5±2.4	2.9±2.4	1.31 (1.14–1.52)	<0.001	1.22 (1.03–1.45)	0.019
Anorexia (mean±SD)	3.2±3.4	1.9±2.5	1.16 (1.04–1.30)	0.006	1.19 (1.04–1.37)	0.008
Dyspnoea (mean±SD)	3.4±2.7	2.2±2.4	1.21 (1.06–1.37)	0.004	1.19 (1.02–1.38)	0.024
Depression (mean±SD)	4.8±3.0	2.6±2.8	1.28 (1.14–1.43)	<0.001	1.28 (1.11–1.47)	<0.001
Nausea (mean±SD)	2.2±2.6	1.2±2.1	1.20 (1.03–1.39)	0.012	1.25 (1.05–1.48)	0.007
Insomnia (mean±SD)	2.3±2.7	1.6±2.1	1.13 (0.99–1.29)	0.074		
Constipation (mean±SD)	4.9±3.7	4.2±3.3	1.06 (0.97–1.16)	0.192		
Dry mouth (mean±SD)	3.3±2.3	2.4±2.5	1.19 (1.03–1.37)	0.013	1.19 (1.01–1.40)	0.032
Overall assessment (mean±SD)	31.2±13.8	21.6±12.7	1.06 (1.03–1.08)	<0.001	1.05 (1.02–1.09)	<0.001

^a^ adjusted for Alb, CRP, and TTR. ^b^ 95% CI: upper and lower limits of the confidence interval with a significance level of 0.05. Alb: albumin; CRP: C-reactive protein; IL: interleukin; IQR: interquartile range; OR: odds ratio; RBP: retinol-binding protein; Tf: transferrin; TLC: total lymphocyte count; TNF: tumor necrosis factor; TTR: transthyretin.
